# Beyond thrombosis: the impact of tissue factor signaling in cancer

**DOI:** 10.1186/s13045-020-00932-z

**Published:** 2020-07-14

**Authors:** Dusten Unruh, Craig Horbinski

**Affiliations:** 1 Department of Neurological Surgery, Northwestern University, 303 East Superior St, Chicago, IL 60611 USA; 2 Department of Pathology, Northwestern University, Chicago, IL 60611 USA

**Keywords:** Tissue factor, Protease-activated receptors, Receptor tyrosine kinases, Integrins, Metastasis, Angiogenesis

## Abstract

Tissue factor (TF) is the primary initiator of the coagulation cascade, though its effects extend well beyond hemostasis. When TF binds to Factor VII, the resulting TF:FVIIa complex can proteolytically cleave transmembrane G protein-coupled protease-activated receptors (PARs). In addition to activating PARs, TF:FVIIa complex can also activate receptor tyrosine kinases (RTKs) and integrins. These signaling pathways are utilized by tumors to increase cell proliferation, angiogenesis, metastasis, and cancer stem-like cell maintenance. Herein, we review in detail the regulation of TF expression, mechanisms of TF signaling, their pathological consequences, and how it is being targeted in experimental cancer therapeutics.

## Introduction

Tissue factor (TF), also named Factor III, CD142, or thromboplastin, is a transmembrane glycoprotein that participates in the rate-limiting step of the coagulation cascade. It derives its name from the ability of tissue extracts from platelets, leukocytes, and organs to clot blood. Morawitz is credited with developing the “classical” theory of blood coagulation in 1905, wherein prothrombin is converted into thrombin when incubated with tissue extracts and calcium [1]. He postulated that components for blood clotting are always present in circulating blood, except for TF (then named thrombokinase), which must be released from tissues [2]. Thirty years later, Howell used the term “Tissue Factor” to describe an extract from tissues that promotes coagulation [3]. TF is constitutively expressed in the subendothelial spaces and adventitia, where it acts as a hemostatic envelope to halt bleeding when it encounters calcium and coagulation factors. TF is abundantly expressed in highly vascularized organs, like the brain, lungs, placenta, heart, and kidneys [4, 5]. During vascular injury, TF is exposed to the blood, where it functions as a cofactor for the circulating zymogen factor VII (FVII). This TF:FVIIa complex can then bind and activate either factor IX (FIX) or factor X (FX), triggering a cascade that generates fibrin and activates platelets, resulting in a hemostatic plug at the site of injury.

TF is highly expressed in many types of cancer [6], and cancer cells can shed TF-active microvesicles (TF-MVs) into the circulation [7]. Not surprisingly, many cancers cause venous thromboembolism (VTE), with high levels of flTF-MVs correlating with increased VTE risk [8]. As a group, cancer patients have an ~ 6-fold increased risk of VTE compared to the general population [9–11]. This paraneoplastic event is called “Trousseau Syndrome,” named after the French physician Armand Trousseau, who was among the first to describe this in the 19th century in his cancer patients [12]. (He also developed VTE himself in 1867 and correctly diagnosed himself with fatal gastric cancer.)

It is now clear, however, that TF has other major roles beyond hemostasis and thrombosis. Ancient organisms like the horseshoe crab (*Limulus polyphemus*) use proteins similar to TF as an immune response to entrap pathogens [13]. Indeed, many components of the mammalian coagulation system share similarities with inflammatory molecules of the immune system, and that, in addition to coagulation, they play important roles in immunity and wound healing [13]. Cancers can use these TF-activated pathways to enhance cell proliferation, cell survival, angiogenesis, metastasis, and cancer stem-like cell (CSC) maintenance. In fact, because of these similarities to wound healing, cancer has been called “the wound that does not heal” [14].

Herein, we present a general overview of the mechanisms controlling TF expression in cancer, discuss TF-mediated signaling pathways, highlight how TF controls cancer progression, and review TF-targeted therapeutic strategies being tested to improve cancer management.

## TF structure and isoforms

**Fig. 1:**
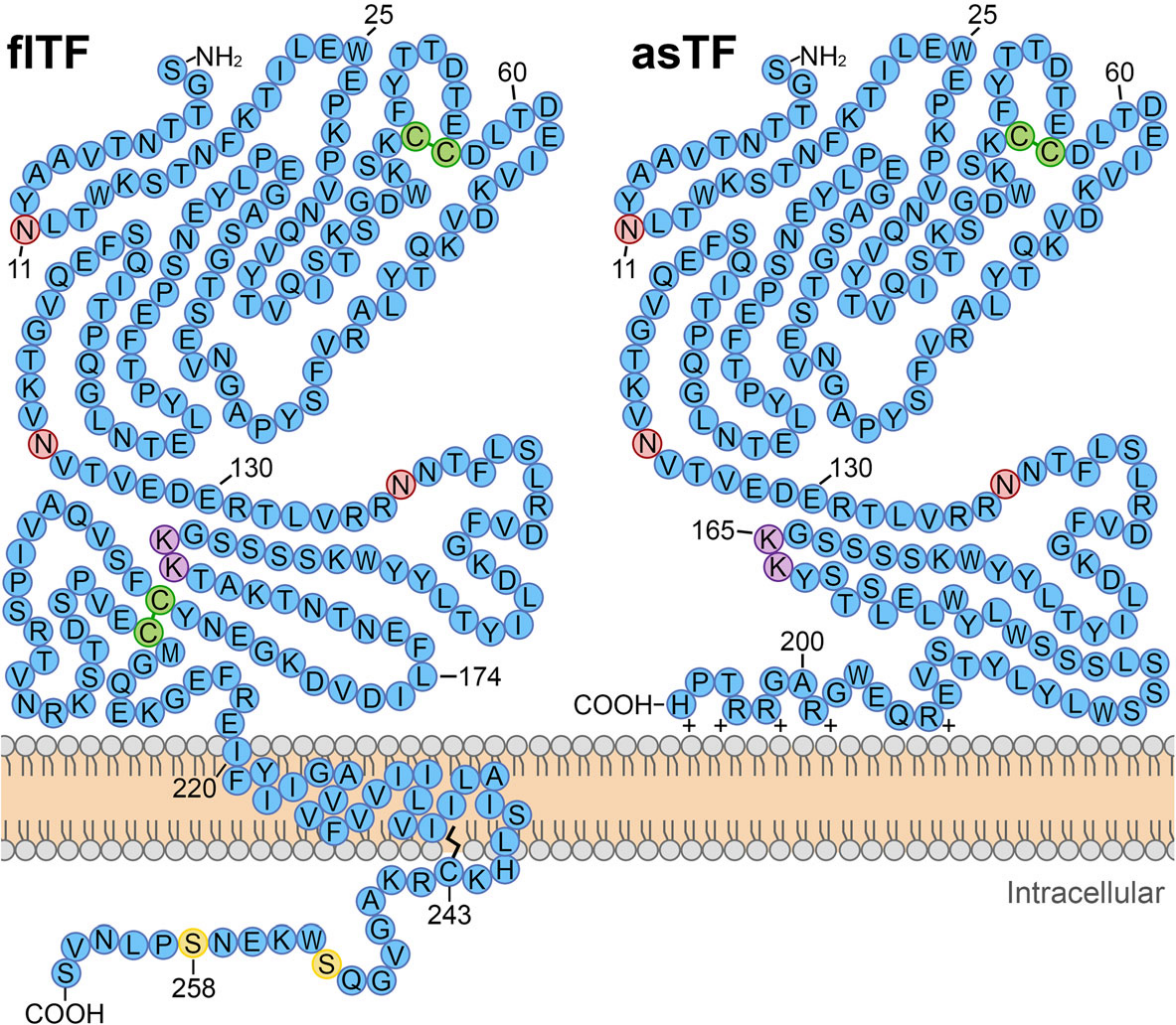
Full length TF (flTF) and alternatively spliced TF (asTF) primary protein structure. flTF is an integral transmembrane protein, whereas asTF lacks a transmembrane and can be secreted. Both isoforms contain the same first 166 residues, which share an N-terminal β-sandwich and the charged lysine doublet (purple circles) necessary for FVII/FVIIa binding. Disulfide bridges are shown with green circles. Glycosylation has been reported to occur on asparagine at sites N11, N124, and N137 (red circles). flTF contains two serine residues in the C-terminus that can undergo phosphorylation (yellow circles). Palmitoylation of C243 is thought to orientate flTF in the plasma membrane. Through alternative splicing of the gene that encodes for TF, *F3*, a frame shift occurs and generates a unique C-terminus, resulting in the formation of asTF. asTF’s unique C-terminus contains a cluster of five positively charged residues (+) that allow asTF to interact with cell membranes

TF is encoded by *F3*, a 12.5 kb gene located on chromosome 1p21.3. This gene contains 6 exons that can produce two different proteins. One protein, the product of all 6 exons, is full-length TF (flTF). The other is an alternatively spliced isoform, termed asTF (Fig. 1). These two proteins have an apparent molecular mass of ~ 47 kDa and ~ 26 kDa, respectively, after sodium dodecyl sulfate-polyacrylamide gel electrophoresis (SDS-PAGE) [15, 16].

Human flTF is a 263 amino acid transmembrane glycoprotein that is comprised of an extracellular domain (residues 1-219), a transmembrane domain (residues 220-242), and an intracellular C-terminal domain (residues 243-263). This portion of TF is structurally homologous to class II cytokine receptors [17, 18]. The extracellular portion of flTF contains two fibronectin type III domains and two disulfide bridges at Cys^49^–Cys^57^ and Cys^186^–Cys^209^ [19]. Although the extracellular domain of TF is structurally similar to the class II cytokine receptors, it does not bind to any cytokines. Instead, it binds to coagulation factor VII (FVII) zymogen. The cytoplasmic C-terminal domain of flTF does not contain any significant homology with other proteins, and it lacks adaptor motifs usually found in the cytokine receptor superfamily that bind with Janus kinase-signal transducer and activator of transcription (JAK-STAT) effectors. Instead, TF’s cytoplasmic C-terminal domain interacts with a variety of scaffolding and adapter proteins that influence cytoskeletal structure and cell signaling.

In 2003, Bogdanov et al. first described asTF, in which exon 5 is spliced out and exons 4 and 6 are fused together. This causes a shift in the open reading frame that produces a unique 40 amino acid C-terminus [15]. The unique C-terminus of asTF lacks a transmembrane domain and is secreted. Although asTF retains a Lys^165^ and Lys^166^ doublet, which is required for TF:FVIIa complex formation, its procoagulant activity is questionable, as it lacks a complete binding site for coagulation factors IX and X [15]. Indeed, asTF has minimal to no procoagulant activity when compared to flTF in vitro [20–22]. However, these studies were primarily conducted in flTF deficient conditions, and asTF may indirectly trigger coagulation [23, 24]. Even so, Ünlü et al. observed that asTF does not affect receptor-ligand kinetics of flTF on endothelial cells [21]. Because of asTF’s positively charged C-terminus, it likely requires a negatively charged phospholipid membrane to promote coagulation, which is often found in cancer.

In addition to alternative splicing, TF can undergo post-translational modifications. The extracellular domain has three N-linked glycosylation sites (Asn^11^, Asn^124^, and Asn^137^), and TF glycosylation may affect the charge of TF and its affinity to FVII [25, 26]. It has widely been assumed that recombinant TF and naturally occurring TF are identical, but Krudysz-Amblo et al. showed that TF derived from the placenta was 5-fold more procoagulant than recombinant TF and that this difference was due to increased glycosylation of placental TF [27]. Phosphorylation is another post-translational modification that affects TF signaling, discussed below. The C-terminus of TF contains two phosphorylation sites (Ser^253^ and Ser^258^) [28]. TF can also undergo palmitoylation, the covalent attachment of fatty acid palmitate to a cysteine (S-palmitoylation) or, less frequently, to threonine or serine (O-palmitoylation) [29]. Palmitoylation controls a wide variety of cellular functions, including protein subcellular trafficking, protein stability, and protein-protein interactions [29]. The intracellular domain of TF has one S-palmitoylation site (Cys^245^) [30], which suppresses phosphorylation of Ser^258^ in the TF C-terminus [31]. However, the extent of TF palmitoylation, and whether it influences plasma membrane microdomain trafficking, is still unclear.

## Noncoagulant signaling by tissue factor

**Fig. 2:**
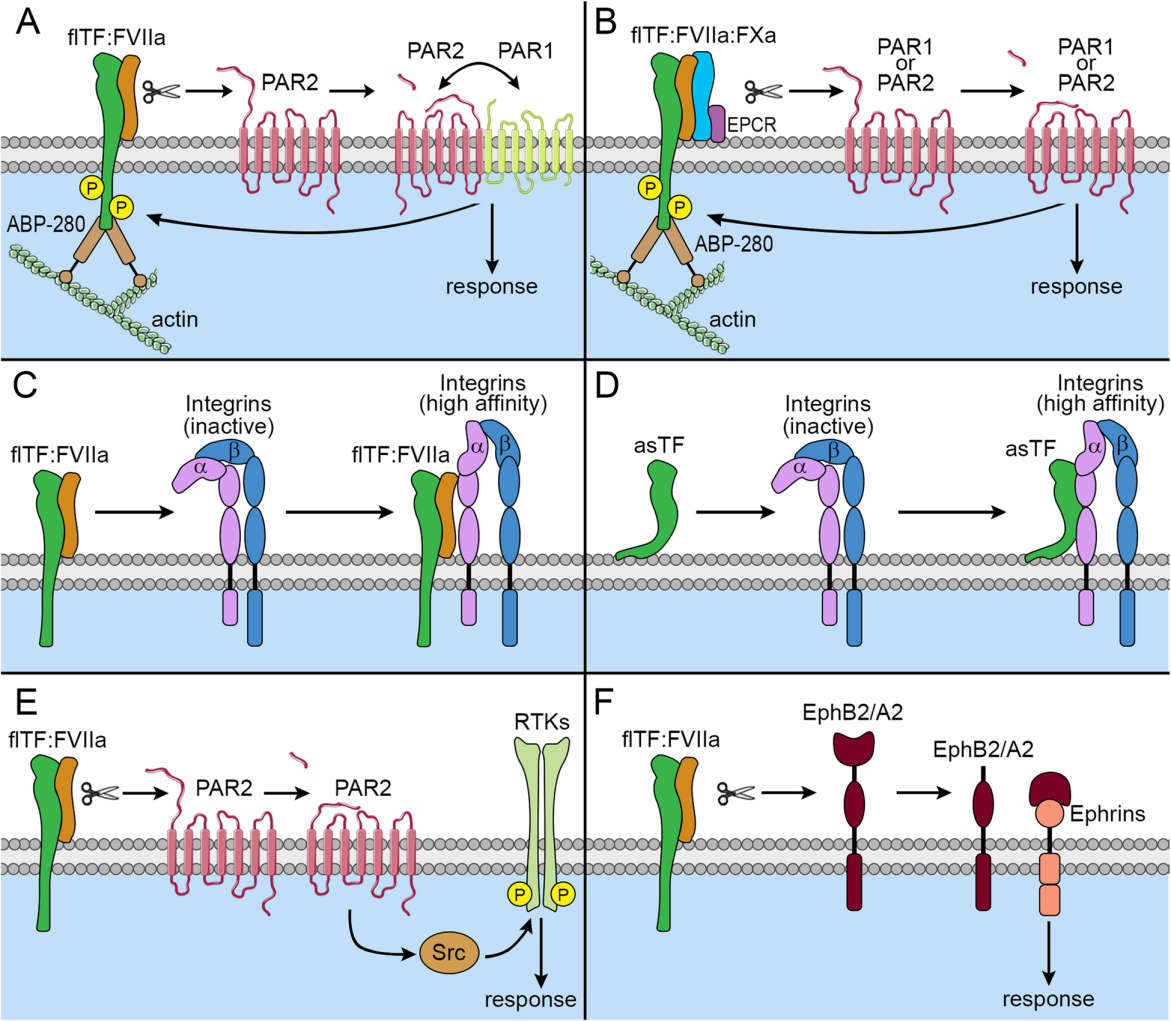
Coagulation independent mechanisms of TF-mediated signaling. **a** flTF:FVIIa can proteolytically cleave PAR2, resulting in the phosphorylation of flTF’s cytoplasmic domain and the recruitment of ABP-280. flTF:FVIIa-mediated cleavage of PAR2 can also promote PAR1/PAR2 heterodimer signaling, when the cleaved tethered ligand binds to adjacent PARs. **b** flTF:FVIIa:FXa can proteolytically cleave both PAR1 and PAR2. **c** Binding of flTF:FVIIa to integrins causes their conformation to switch to a high affinity state. **d** asTF can bind to integrins independent of FVII, causing a conformation switch. **e** flTF:FVIIa induces transaction of RTKs in a PAR2- and Src-dependent manner. **f** flTF:FVIIa proteolytically cleaves both EphB2 and EphA2, which then can signal through ephrins

Although TF is classified as a cytokine class II receptor, its signaling mechanism differs greatly from that family of receptors. TF can trigger both proteolytic and non-proteolytic cell signaling, and both TF isoforms have unique mechanisms of action (Fig. 2). On tumor cells, flTF binds the inactive zymogen, FVII, forming a flTF:FVII complex in which FVII is rapidly converted into an active protease, FVIIa. The flTF:FVIIa complex then binds and activates FX, creating a ternary flTF:FVIIa:FXa complex. This complex cleaves several protease-activated receptors (PARs), which belong to the seven-transmembrane G protein-coupled receptor family whose members are irreversibly activated by proteolytic cleavage of the N-terminus. Cleavage of the N-terminus on PAR releases its tethered ligand, allowing it to fold back on itself and bind to the extracellular portion of loop 2, in turn altering the binding affinity of PAR to intracellular heterotrimeric G proteins. These G proteins are composed of three unique subunits, α, β, and γ, and act as molecular switches inside cells to initiate a remarkably diverse array of functions.

To date, the PAR group of receptors consists of PAR1, PAR2, PAR3, and PAR4. TF activation of the coagulation cascade generates thrombin, and PAR1 was first discovered in 1991 as a receptor for thrombin on platelets [32]. PAR1, PAR3, and PAR4 are all activated by thrombin; PAR2 is the only PAR that is not [33]. The flTF:FVIIa:FXa complex can directly cleave both PAR1 and PAR2, and flTF:FVIIa can cleave PAR2. Signaling by the flTF:FVIIa:FXa complex is supported by the endothelial protein C receptor (EPCR), enabling PAR signaling, and is required for the induction of proinflammatory genes [34, 35]. In contrast, flTF:FVIIa elicits MAPK signaling through PAR2, not PAR1, and also promotes calcium mobilization, activation of Src family kinases, and activation of other pathways like PI3K, JAK/STAT, and β-catenin [36–43]. However, studies show that PARs often exist in close proximity to each another and are capable of forming homodimers and heterodimers [44]. Furthermore, cleaved PAR2 can transactivate PAR1, and vice versa [44, 45]. Thus, both PAR1 and PAR2 signaling are closely linked, and can potentially lead to each other’s activation by proxy.

Once PARs are cleaved, they can transactivate nearby membrane-bound receptor tyrosine kinases (RTKs) [46]. Such transactivation could occur via one of two mechanisms: (i) ligand-independent recruitment of non-tyrosine kinases; (ii) a “triple membrane passing signal” mechanism wherein membrane-bound matrix metalloproteases are activated, leading to receptor ligand shedding, in turn causing paracrine and/or autocrine RTK activation [47]. flTF:FVIIa cleavage of PARs causes transactivation of multiple RTKs, including EGFR, PDGFRβ, and IGF-1R [48–51]. Because this generally happens within minutes, and does not depend on secreted ligand, flTF:FVIIa transactivation of RTKs through PARs is mostly via the former, ligand-independent mechanism. In fact, flTF:FVIIa activation of PAR2 leads to RTK phosphorylation via a ligand-independent, Src-dependent intracellular pathway [48–51]. This means that even when extracellular ligands for RTKs are absent or when cells are in the presence of extracellular RTK inhibitors, flTF:FVIIa may still activate those RTKs through PAR2. Even though intracellular activation of RTKs via flTF may confer resistance to RTK small molecule inhibitors, anti-RTK antibodies are often still clinically effective [52].

A lot of TF’s non-coagulant effects are dependent on integrins. These are heterodimeric transmembrane receptors that function as adhesion molecules for both cell-cell and cell-extracellular matrix (ECM) interactions, and mediate a variety of biological processes that include survival, proliferation, stemness, and migration [53]. There are 24 integrins, each being composed of various combinations of α and β subunits. Integrins can assume three conformation states, including bent (inactive), extended (intermediate affinity), or extended with open conformation (high affinity) [54]. flTF directly interacts with α3, α6, and β1 integrin subunits [55, 56], and flTF:FVIIa associates with β1 subunits in the active conformation, thus inducing the internalization of the TF:FVIIa-β1 integrin complex via GTPase Arf6 [57]. Although PAR2 activation enhances the affinity of flTF:FVIIa to β1 integrins, blocking PAR2 activation is not sufficient to prevent flTF:FVIIa binding to β1 integrins [56].

In addition to flTF:FVIIa cleaving PAR2, it also cleaves receptors in the Eph RTK family [58]. The Eph RTK family includes 14 members separated into nine EphA and five EphB receptors, that govern cell proliferation, differentiation, and mobility [59, 60]. flTF:FVIIa directly cleaves both EphB2 and EphA2, independent of PAR2 activation or the flTF:FVIIa:FXa ternary complex [58]. The consequences of this are the subject of ongoing investigation.

The flTF cytoplasmic domain plays an especially important role in non-coagulant signaling. It serves as a scaffold for molecules involved in adhesion, migration, and cytoskeleton organization, such as actin-binding protein-280 (ABP-280). TF localizes to lamellipodia, which are created by cytoskeletal protein actin projections on the invasive edges of cells [61–63]. TF binding of ABP-280 may also positively regulate its release via flTF-MVs [64], resulting in paracrine signaling [65]. The flTF cytoplasmic domain is phosphorylated at Ser253 by protein kinase C (PKC) and at Ser258 by p38 MAPK [28, 31, 36, 66]. Such phosphorylation promotes the physical association of flTF with α3β1 integrins, thereby enhancing cell migration on laminin 5 [55]. This flTF cytoplasmic domain interacts with the PI3K regulatory subunit p55 and is required for flTF-induced interleukin-8 (IL-8) expression and resultant angiogenesis [57]. The cytoplasmic domain of flTF does not affect the procoagulant activity of flTF, nor does it alter the proteolytic activation of PAR2 by flTF:FVIIa [57, 67–69]. However, once the flTF cytoplasmic domain is phosphorylated, it inhibits PAR2-dependent angiogenesis [55, 70]. Interestingly, mice lacking the flTF cytoplasmic domain do not die in utero, unlike TF-deficient mice, and flTF:FVIIa association with β1-integrin does not require the cytoplasmic domain [57, 71].

Whereas flTF can either remain anchored within the cell membrane or be secreted, asTF lacks the transmembrane domain and is only secreted. Although asTF is not very pro-coagulant, it does have non-hemostatic functions. For example, asTF is a powerful pro-angiogenic molecule via non-proteolytic ligation of both α6β1 and αVβ3 integrins, leading to increased FAK, PI3K/AKT, and MAPK signaling [16, 72]. In contrast to flTF, asTF can bind integrins without the help of FVII [16].

## Regulation of tissue factor expression

TF is constitutively expressed on a wide variety of cells, especially in the perivascular niche, where it surrounds the abluminal side of the blood vessel, ensuring that clot formation happens quickly once blood escapes the endothelium [4, 73]. Endothelial cells that are normally in contact with blood express very little TF at baseline, though certain stimuli can trigger its upregulation, like bacterial lipopolysaccharide [74, 75]. Several miRNAs, including miR-181b, miR-19, miR-20a, miR-93/106b, and miR-520 g, can bind to the *F3* transcript and limit its translation into TF [76–80]. PARP-14 is another posttranscriptional regulator of TF expression that reduces TF RNA levels by forming a complex with the mRNA-destabilizing protein tristetraprolin [81].

Multiple growth factors and cytokines induce TF expression. For example, hepatocyte growth factor (HGF) upregulates TF by activating Met in a Src kinase-dependent manner [82]. Other growth factors, including basic fibroblast growth factor (bFGF), platelet-derived growth factor (PDGF)-BB, PDGF-CC, PDGF-AA, bone morphogenetic protein-7 (BMP-7), transforming growth factor-β (TGF-β), and vascular endothelial growth factor (VEGF) can also increase TF [83–90]. During inflammation, TF can be upregulated by a variety of cytokines and signaling molecules, including interferon-γ (IFN-γ), tumor necrosis factor-α (TNF-α), IL-6, IL-1β, IL-33, and histamine [91–99]. Activated T lymphocytes release CD40-ligand (CD40L), which also induces TF [100]. Conversely, the anti-inflammatory cytokines IL-4, IL-10, and IL-13 all suppress TF [101–103].

**Fig. 3:**
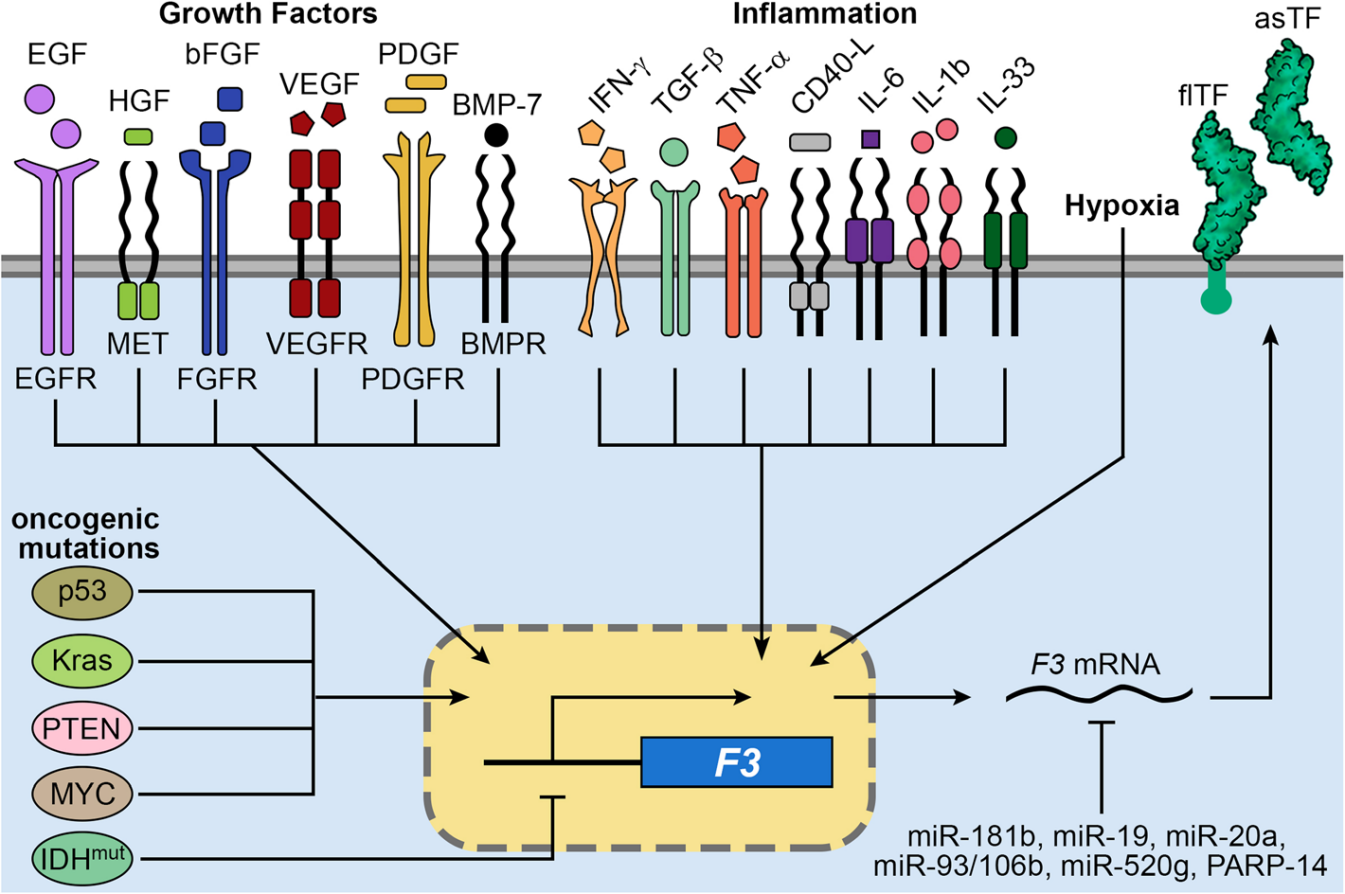
Induction of tissue factor expression in cancer. Growth factors, inflammation, hypoxia, and oncogenic signaling mechanisms activate signaling pathways that drive the expression of TF. Conversely, TF is downregulated by some micro RNAs (miRs), and by hypermethylation induced by IDH^mut^

In cancer, TF expression can be directly driven by pro-oncogenic events (Fig. 3). For example, mutations in the tumor suppressor *TP53* and proto-oncogene *KRAS* activate MAPK and PI3K/AKT signaling pathways, in turn stimulating TF expression [104, 105]. In glioma, amplification of *epidermal growth factor receptor* (*EGFR*) and/or its constitutively active mutant form, EGFRvIII, promotes the expression of TF, PARs, and ectopic synthesis of FVII, thus sensitizing the cells to TF-mediated signaling [106]. EGFR-mediated TF expression depends on activator protein-1 (AP-1) and is associated with c-Jun NH(2)-terminal kinase (JNK) and JunD activation [107]. Blocking EGFR signaling in human carcinoma and glioma cells diminishes TF expression [108]. Loss of the tumor suppressor Pten or E-cadherin also leads to TF upregulation [109–111]. In neuroblastoma, *MYCN* amplification positively correlates with TF [112].

Cancer-associated hypoxia also stimulates TF expression via canonical hypoxia-associated signaling molecules, including hypoxia-inducible factor 1-alpha, early growth response gene-1 (Egr-1), Cyr61, and VEGF [113, 114]. In glioblastoma (GBM), hypoxia is sufficient to increase TF production in cultured GBM cells, and tumor cells surrounding necrotic, hypoxic zones stain strongly for TF [109, 115].

In contrast, we found that gliomas with mutations in *isocitrate dehydrogenase 1* or *IDH2* (collectively “IDH^mut^”) hypermethylate the early coding region of *F3* and suppress its transcription, correlating with less flTF-MV production, less risk of VTE, and less aggressive behavior [116, 117]. This appears to be unique to IDH^mut^ gliomas, as other IDH^mut^ cancers, like cholangiocarcinoma and acute myeloid leukemia, neither hypermethylate *F3* nor suppress TF production [51, 116].

## Pathophysiological effects of TF signaling

**Fig. 4:**
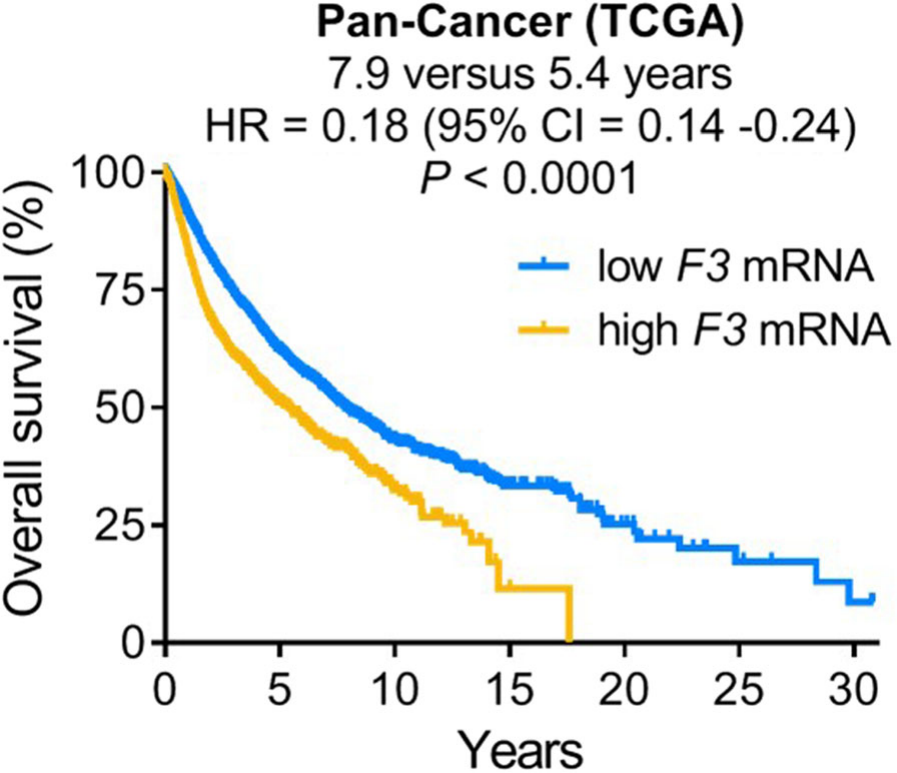
High *F3* mRNA correlates with worse prognosis in cancer. Overall survival of Pan-Cancer TCGA cancer patients, stratified according to *F3* mRNA via Cutoff Finder (http://molpath.charite.de/cutoff/). *N* = 8556 for low *F3* mRNA and *N* = 2288 for high *F3* mRNA

While TF clearly has critical roles to play in normal hemostatic and non-hemostatic cell functions, such activities can also greatly contribute to the malignant behavior of cancer. Elevated TF is a common feature of many cancers, and generally correlates with worse patient survival (Fig. 4) [6]. Here, we will discuss some of the key studies examining the pathological consequences of TF expression in cancer, including effects on angiogenesis, invasion, cell survival, metastasis, and maintenance of cancer stem-like cell (CSC) populations.

### Tumor angiogenesis

flTF:FVIIa activation of PAR2 triggers downstream transcription of several proangiogenic genes, including *VEGF*, *IL8*, *FGF2*, and *CXCL1* [38, 118–120]. The cytoplasmic domain of flTF is important for controlling VEGF expression, and its phosphorylation correlates with VEGF expression in cancer [121–123]. Consistent with this, flTF deletion results in vascular failure during embryogenesis, and just deleting the flTF cytoplasmic domain is sufficient to suppress VEGF production [121, 122, 124]. Of note, although flTF deletion in mice results in vascular abnormalities, it does not phenocopy VEGF deletion [125]. Positive correlations between flTF and vascular density have been reported in cancers of the brain, pancreas, prostate, large bowel, lung, and breast [126–131]. This seems to be primarily through PAR2, because although some have also implicated PAR1 in blood vessel growth, PAR1-deficient mice show normal wound healing and postnatal angiogenesis [132–135]. Studies in PAR1- and PAR2-deficient mice further support the notion that signaling through PAR2, but not PAR1, is important for tumor angiogenesis [136].

Like flTF, elevated expression of asTF increases the expression of multiple genes that promote angiogenesis like *VEGF*, *AREG*, and *EFEMP1* [23], and at least part of asTF-induced cancer angiogenesis is dependent on ligation of β1 and β3 integrins [16, 137]. Furthermore, asTF does not require FVIIa to induce angiogenesis [16]. High expression of asTF correlates with worse patient survival in lung and gastric cancer [138, 139].

### Proliferation, cell survival, and apoptosis

Both flTF and asTF can increase the proliferation of many different kinds of cancer, though perhaps by different mechanisms [140]. In colon cancer cells, flTF:FVIIa activates PAR2, leading to increased proliferation through the PKCα and ERK1/2 pathways [141, 142]. In breast cancer cells, the pro-proliferative effect of asTF depends on β1 integrin [143]. However, the response of cancers to TF likely depends on their molecular background. For example, we found that GBM cells with driver mutations in *EGFR* are far more dependent on endogenous TF for cell proliferation than GBM cells with driver mutations in *NF1* [116].

flTF has a number of anti-apoptotic effects in cancer cells, including activation of PI3K, AKT, and JAK/STAT5 signaling pathways; suppression of death-associated protein kinase-1; and upregulation of Bcl_XL_ [144–148]. As a result, flTF can prevent anoikis (Greek for “homeless”), a form of apoptosis that is induced by the lack of anchorage-dependent survival signaling caused by loss of surrounding extracellular matrix [143, 146, 149].

### Cancer stem-like cells

Special subpopulations of cells within cancer are capable of self-renewal. These cells are commonly referred to as cancer stem-like cells (CSCs), and they contribute to therapy resistance, recurrence, and distal metastasis [150]. Our data, and that of others, indicate that TF contributes to the CSC phenotype [151]. For example, TF is positively correlated with well-known markers of CSC like CD44 and CD133, and is enriched on CSCs compared to cancer cells without self-renewing capacity [152–156]. In conditional knock-down experiments, lack of TF can prevent the growth of some xenografted cancers for prolonged periods of time, followed by abrupt and rapid growth when TF expression is restored [157]. Conversely, others found that TF may not be a regulator of CSC populations in all cancer types. Schaffner et al. showed that EPCR negative cells and receptor blocking EPCR antibodies reduced breast cancer stem cell populations, whereas TF expression did not alter the CSC phenotype [158].

### Invasion and metastasis

Metastasis is a Greek word meaning “displacement” and is the result of multiple cellular processes working together to allow cancer cells to invade normal tissue beyond the original tumor site, survive while traveling through the blood or lymphatic system, and form a new tumor in a distant site. TF is prominently expressed at the invasive edge of tumors and positively correlates with invasiveness in cancers from a variety of organs, including the liver, breast, pancreas, and lung [159–162]. TF expression is also up to 1000-fold greater in metastatic cells than in non-metastatic cells [163]. An unbiased shRNA screen showed that TF is one of the most powerful drivers of metastasis in osteosarcoma [164]. Such correlative findings have been verified by functional studies in vivo [23, 163, 165].

The mechanisms underpinning TF’s effects on invasion and metastasis mostly resemble those already noted in other pro-malignant functions. The pro-invasive effects of flTF on cancer cells appear to depend on the cytoplasmic domain, as expression of mutant flTF constructs that lack the cytoplasmic domain are far less metastatic [166, 167]. The cytoplasmic domain of flTF promotes invasion by activating the GTPase Rac1 and p38 MAPK, which interact with actin filaments and cause extension of filopodia and lamellipodia [61]. flTF-mediated activation of PAR2 also recruits actin-binding protein to the cytoplasmic domain of flTF, and promotes cytoskeleton reorganization and cell motility by activating MAPK and cofilin pathways [62, 63, 168]. Activated PAR2 stabilizes and increases β-catenin expression [169], and in glioma, the pro-invasive effects of flTF:FVIIa is dependent in part on β-catenin [116]. However, flTF-β1 integrin interaction promotes cell migration when the flTF cytoplasmic domain is phosphorylated [55], suggesting the requirement of PAR2 activation. In contrast, asTF can increase cell motility by directly binding and activating β1 integrins [170].

The release of flTF-MVs by tumors can also promote metastasis via paracrine signaling within the tumor microenvironment and at distal sites. flTF-MVs are elevated in the plasma of cancer patients [171, 172] and can activate PAR1 and PAR2 on nonmalignant cells [173, 174]. Endothelial cells that are stimulated with flTF-MVs express increased adhesion molecules and secrete pro-inflammatory molecules that can recruit pro-tumor monocytes, establishing a pre-metastatic niche for circulating cancer cells [173, 175].

In addition to the aforementioned suppression of anoikis, flTF-mediated activation of the coagulation pathway is important once cancer cells enter the bloodstream. Two monoclonal inhibitory antibodies were developed to study the role of blood coagulation in metastasis. The first, mAb-10H10, blocks flTF:FVIIa-mediated PAR2 activation without any significant effect on coagulation. The second, mAb-5G9, blocks the formation of the flTF:FVIIa:FXa complex, thereby inhibiting stimulation of both PAR1 and PAR2, as well as impairing coagulation [176]. Using both antibodies in a model of patient-derived breast cancer xenografts, Versteeg et al. showed that, whereas mAb-10H10 inhibited primary tumor growth, mAb-5G9 inhibited its metastatic spread [56]. Palumbo et al*.* suggested that flTF further supports tumor metastasis via thrombin-dependent and platelet-dependent mechanisms that prevent natural killer cell clearance of cancer cells [177]. In sum, both TF-mediated thrombin generation and TF-mediated intracellular signaling contribute to distal metastasis.

## Tissue factor as a target for cancer therapy

Because TF plays multiple roles in the malignant behavior of cancer, blocking its signaling might improve cancer treatment. A direct inhibitor of TF:FVIIa, recombinant nematode anticoagulant protein c2 (rNAPc2), has been shown to reduce tumor growth in preclinical models of colorectal cancer and melanoma [178, 179]. These findings promoted a clinical trial to test the safety of rNAPc2 to treat colon cancer (ClinicalTrial.gov, ID NCT00443573); however, this study was suspended, and no results were published. rNAPc2 has a narrow therapeutic window, with a high risk of bleeding complications [180], and this could likely explain why the rNAPc2 trial was suspended. Although directly targeting TF disrupts normal hemostasis and increases risk of uncontrollable hemorrhage, there are ways of blocking non-hemostatic aspects of TF activity. For example, the clinical grade formulation of the mAb-10H10 (CNTO 2559) does not interfere with hemostasis and has inhibited breast cancer growth in mice [181].

As discussed above, one of the primary targets of flTF is the PAR signaling nexus. Because flTF activates PARs independently of its role in coagulation, targeting PARs could limit many of the pro-cancer effects of flTF without the dangerous side effects of direct flTF inhibition. Small molecule PAR inhibitors have therefore been tested as potential cancer therapeutics. The PAR2 antagonist, GB88, and its derivatives, block PAR2 activation and reduced inflammation, yet these inhibitors have low IC_50_ and solubility, limiting their effectiveness in vivo [182–184]. Furthermore, for unclear reasons, GB88 blocks only certain PAR2-mediated signaling pathways, such as Ca^2+^ mobilization, but not MAPK signaling [185]. Recently, a new PAR2 antagonist, I-191, was developed that has a much higher IC_50_ and solubility and blocks both Ca^2+^ mobilization and MAPK signaling [182]. Two PAR1 inhibitors exist, atopaxar and vorapaxar, and both inhibit cancer progression in preclinical models [186]. An additional approach for blocking PAR signaling involves the use of cell-penetrating peptide antagonists termed “pepducins” [187]. PAR1-targeting pepducin P1pal-7 blocked lung cancer cell migration and inhibited lung tumor growth by 75% [188]. For excellent in-depth reviews on targeting PARs, see Ramachandran et al. and Hamilton et al. [185, 189]. However, while PAR inhibitors seem to be well-tolerated in animal models, it remains to be determined whether targeting PAR1 or PAR2 will prove effective in cancer patients.

flTF requires FVII and FX complex formation for efficient proteolytic signaling. Because FVII and FX are vitamin K-dependent, their expression can be reduced by using vitamin K antagonists, like warfarin, which has been shown to suppress tumorigenesis [190–192]. Warfarin also blocks the growth and spread of pancreatic cancer by inhibiting signaling of another RTK, Axl [193]. An FXa inhibitor, rivaroxaban, did not directly affect the growth of TF-positive pancreatic cancer xenografts in immunodeficient mice [194], but did suppress tumor growth in an immunocompetent setting that was partially dependent on reprograming tumor-associated macrophages [195]. A selective small molecule inhibitor of FVIIa, PCI-27483, inhibited tumor growth in preclinical models [196], although combining it with gemcitabine was not clearly superior to gemcitabine alone in a phase 2 clinical trial against pancreatic cancer [197].

Because TF is highly expressed on the surface of many kinds of cancer cells, some have investigated the ability of TF-specific antibody:drug conjugates to deliver tumoricidal drugs. One prominent example is a human anti-TF antibody conjugated to the cytotoxic agent monomethyl auristatin E (TF-011-MMAE) [198]. TF-011-MMAE has suppressed tumor growth and metastasis in a variety of preclinical cancer models, even those expressing relatively low amounts of TF, without significant effect on coagulation, and showed better intracellular internalization than antibody:drug conjugates targeting EGFR or HER2 [198–200]. TF-011-MMAE, now renamed HuMax-TF-ADC, has undergone multiple phase 1 trials demonstrating its safety and is currently undergoing a multicenter phase 2 clinical trial for safety and efficacy against cervical cancer and solid tumors (ClinicalTrial.gov, ID: NCT03438396, NCT03485209).

Hu et al. have developed chimeric antibody-like homodimers that consist of FVII fused to the Fc domain of IgG1 [201]. These immunoconjugates, which are named “ICONs,” target TF-expressing tumors, and induce both antibody-dependent cellular cytotoxicity and complement-dependent cytotoxicity [202–204]. ICONs lack procoagulant activity due to the elimination of Lys^341^, and when administered intravenously, inhibit the growth of melanoma, prostate, and head and neck tumors [201, 202, 205]. Because first generation ICONs are relatively large molecules (210 kDa), their bioavailability within solid tumors is questionable. A smaller (100 kDa), more efficient second-generation ICON, L-ICON1, is now being evaluated in preclinical studies [206].

A rapidly emerging immunotherapy strategy for treating cancer is chimeric antigen receptor (CAR)-modified T cells (CAR T) [207, 208]. To date, CAR T therapy against hematopoietic cancers has met with some success [209], but whether it has real potential against solid tumors is debatable. One limiting factor of CAR T in solid tumors is finding suitable antigens to target [207, 208]. Zhang et al. developed human T cells modified to express the light chain of mouse FVII, since it binds strongly to both human and mouse TF [210]. These cells, termed TF-CAR T cells, reduced tumor growth and metastasis in NOG mice with human lung cancer xenografts [210]. However, off-target toxicity is one of the main limiting factors of CAR T therapy, so although the authors found no toxicity in mice from the TF-CAR T cells [210], it still remains to be determined whether this approach will have any off-target toxicity in humans.

Since the secreted TF isoform, asTF, also plays a prominent role in cancer malignancy but is only minimally procoagulant, it can be directly targeted without worrying about bleeding. Bogdanov et al. developed an asTF-specific neutralizing antibody termed “RabMab1” [15, 143, 170]. RabMab1 is capable of blocking asTF from binding to integrins and inhibits pancreatic and breast cancer growth in vivo [143, 170]. Other than RabMab1, asTF-specific therapies are scarce. However, other asTF-directed approaches could potentially include inhibitory RNAs that specifically target the unique exons 4-6 spliced sequence or by blocking splicing regulatory proteins that produce asTF [211].

## Conclusion and future perspectives

It is now clear that TF has a wide range of activities beyond thrombosis, and that these activities are utilized by cancers to increase their malignancy. Further insights into TF signaling, and how to block pro-cancer effects while retaining its hemostatic capacity, may lead to more effective therapies against the pro-tumor effects of TF.

## Data Availability

Not applicable for this review

## Abbreviations

asTF: Alternatively spliced tissue factor
bFGF: Basic fibroblast growth factor
BMP: Bone morphogenetic protein
CAR T: Chimeric antigen receptor-modified T cells
FVII: Coagulation factor VII
FIX: Coagulation factor IX
FX: Coagulation factor X
CSC: Cancer stem-like cells
EGFR: Epidermal growth factor receptor
EPCR: Endothelial protein C receptor
flTF: Full-length tissue factor
GBM: Glioblastoma
HGF: Hepatocyte growth factor
ICONs: Immunoconjugates
INF-γ: Interferon-γ
MVs: Microvesicles
rNAPc2: Nematode anticoagulant protein c2
PAR: Protease-activated receptor
PKC: Protein kinase C
PDGF: Platelet-derived growth factor
RTKs: Receptor tyrosine kinases
TF: Tissue factor
TGF-β: Transforming growth factor-β
TNF-α: Tumor necrosis factor-α
VEGF: Vascular endothelial growth factor
VTE: Venous thromboembolism
